# Experimental and Analytical Framework for Predicting Nonlinear Viscoelastic–Viscoplastic Behavior of Polymers

**DOI:** 10.3390/polym17233095

**Published:** 2025-11-21

**Authors:** Alen Oseli, Matic Šobak, Lidija Slemenik Perše

**Affiliations:** Laboratory of Experimental Mechanics, Faculty for Mechanical Engineering, University of Ljubljana, Aškerčeva ulica 6, 1000 Ljubljana, Slovenia; alen.oseli@fs.uni-lj.si (A.O.); matic.sobak@fs.uni-lj.si (M.Š.)

**Keywords:** polymers, linear viscoelastic behavior, nonlinear viscoelastic behavior, viscoplastic behavior, Schapery’s nonlinear model

## Abstract

The present research addresses the modeling of viscoelastic–viscoplastic behavior of polymers with a theoretical expansion of Schapery’s nonlinear viscoelastic model by incorporating two components of irrecoverable processes, displaying material flow and viscoplastic behavior (structure- and load-related irrecoverable process). The theory is accompanied by an experimental and analytical framework for identifying model parameters. Introduced multi-scale analysis allows evaluation of pure linear and nonlinear viscoelastic, as well as viscoplastic behavior, enabling the study of their contribution to overall material response. Model performance was examined with creep recovery tests on two versatile and well-established thermoplastic polymers with different morphological structures: amorphous ABS exhibiting notable flow and semi-crystalline POM, where flow may be neglected. Results show extremely accurate predictions and exceptional agreement with experimental data, as the error was found to be less than 5% ranging from infinitesimally small to relatively high loading magnitudes (from 0.1 to 15 MPa of shear stress) at 70 °C (maximum operating temperature). Notably, viscoplastic strains were detected even within linear viscoelastic domain, suggesting that these effects are not related to yield phenomena (associated with progressive/damaging mechanisms), but rather provide an explanation for the material’s inability to fully recover. With its predictive capability and adaptability, the model demonstrates to be a powerful tool for capturing realistic material responses not only for the considered but also applicable to other molecular systems.

## 1. Introduction

Sustainability of polymers and polymer-based structures presents a major challenge addressed by numerous initiatives and directives in the EU and worldwide, as their development move towards complex geometries, extreme utilization, lower material consumption, etc., [1,2]. Although the sustainability of such structures may be evaluated in several ways, predicting their behavior in a virtual world offers several advantages (generally related to time, cost, and energy efficiency) over real-time testing [3,4]. Currently, the considered linear as well as nonlinear viscoelastic laws are deemed inadequate to provide accurate theoretical or numerical predictions [5,6]. While they are sufficient to deliver reliable material response over a broad range of external excitations (up to high stress–strain states), they are unable to determine residual deformations [7,8]. In numerous instances, their accumulation leads to premature functional or structural failure, and consequently substantial reduction in the structure’s operational lifetime. Accordingly, it is of key importance to update the existing laws and models with integration of components capable of evaluating those permanent deformative mechanisms, which was the main focus of the presented research.

Currently available models are based on the theoretical framework of the phenomenological theory of viscoelasticity. The theory is founded on two main conditions, i.e., proportionality and additivity between cause and response, formulating Boltzmann superposition, which describes the linear viscoelastic response of polymers [9,10]. As soon as one of the conditions collapses, the material exhibits nonlinear viscoelastic behavior [6,11]. Although the transition from linear to nonlinear occurs gradually [12,13,14], an arbitrary value determines the linear viscoelastic limit (stress or strain) where the material property deviates (from 3 to 5%) from the predicted linear one [15]. It primarily depends on selected material, loading (extension, shear, etc.) [16,17] and environmental (temperature, moisture, etc.) conditions, as well as other parameters (particles, concentration, etc.) [15,18,19].

For the subsequent nonlinear viscoelastic behavior, various models are available. In contrast to semiempirical ones (power-law, single integra, etc.) [6], models founded and emerged from linear viscoelastic theory provide insight into the underlying nonlinear mechanisms through stress- or strain-related dependencies, such as Knauss-Emri [20] and Schapery’s [21] nonlinear model. While the Knauss-Emri model describes nonlinear viscoelastic behavior through changes in free volume [22,23], Schapery’s model describes such behavior through nonlinear parameters representing a third- or higher-order dependencies of Gibbs free energy (potential energy for molecular reconfiguration) [21]. Although both models offer accurate predictions of nonlinear viscoelastic response when material is loaded, they are unable to evaluate residual stress–strain state upon releasing the load [7,8]. Besides analytical and experimental simplicity as well as apparent numerical integration, the prevailing implementation of Schapery’s model is mainly related to its flexibility and integrative ability to support numerous modifications and expansions, which are crucial for the task at hand.

There are various forms of modifications and expansions of Schapery’s nonlinear model evaluating types of loading types and inputs [21,24,25,26,27], environmental effects [28,29,30], aging effects [31], material anisotropy [32], and most importantly also those, who cover irrecoverable processes, such as material flow [33] and viscoplastic effects [7,8,34,35,36,37,38,39]. Models, covering material flow, portray irrecoverable deformation resulting from inherent molecular structure designed for amorphous polymers under extreme temperatures [33], while models, covering viscoplastic effect, demonstrate irrecoverable deformation resulting from excessive loading conditions for all other polymer types [34,35,36,37,38,39]. A linearized formulation of viscoplastic behavior (progressive process) was first introduced by R. A. Schapery [7,8]. However, various research works suggested exponential or other formulations to portray damaging as well as diminishing viscoplastic processes [34,35,36,37,38,39]. Nevertheless, while models dealing with material flow lack viscoplastic contribution, the models addressing viscoplastic behavior need integration of material flow.

Hence, the aim of the presented research was to extend Schapery’s nonlinear viscoelastic model by introducing two components of irrecoverable deformation, displaying material flow (structure-related irrecoverable process) and viscoplastic behavior (load-related irrecoverable process). Moreover, the modification of Schapery’s model was accompanied by an experimental and analytical framework for model evaluation. Such formulation would enable predictions of nonlinear Viscoelastic–Viscoplastic behavior for all types of polymers, covering various permanent deformative mechanisms from nonlinear material flow to diminishing and damaging viscoplastic phenomena.

## 2. Theoretical Part

### 2.1. Nonlinear Viscoelastic–Viscoplastic Behavior of Polymers

Within this section, Schapery’s nonlinear viscoelastic model is addressed along with expansions portraying irrecoverable deformations, namely, flow and viscoplastic component. Such behavior may be formulated in many forms, concerning various types and modes of loading [21,25]; however, shear creep behavior (change in geometry) is considered, as its nature coincides with theoretical definitions and experimental evaluations of irrecoverable effects [33,40]. According to R. A. Schapery, the generalized nonlinear viscoelastic response in shear, i.e., the time-dependent shear strain—$\gamma \left(t\right)$ for an arbitrary external load, i.e., shear stress—τ, is expressed as [21]:

(1) $$\gamma_{nve}\left(t\right)=g_{0}J_{0}\tau +g_{1}\int_{0}^{t}∆J\left(\psi -\psi^{\prime }\right)\frac{d\left(g_{2}\tau \right)}{d\rho }d\rho ,$$

and

(2) $$\begin{array}{c} \psi \equiv \int_{0}^{t}\frac{dt^{\prime }}{a_{\tau }\left[\tau \left(t^{\prime }\right)\right]}\;for\;a_{\tau }<0,\\ \psi^{\prime }\equiv \psi \left(\rho \right)\equiv \int_{0}^{\rho }\frac{dt^{\prime }}{a_{\tau }\left[\tau \left(t^{\prime }\right)\right]}, \end{array}$$

where ψ represents the internal or reduced time, ρ is an integral time variable, $J_{0}$ instantaneous shear creep compliance portraying the elastic response of the material, $∆J\left(t\right)$ transient shear creep compliance depicting the linear viscoelastic response of the material, and $g_{i}\left(\tau \right)$ with $a_{\tau }\left(\tau \right)$ stress-dependent nonlinear parameters or nonlinear contributors to the linear viscoelastic response reflecting the third or higher order dependencies of the Gibbs free energy (potential energy for molecular reconfiguration) [7,8,21]. The above parameters may be understood as: nonlinear contribution to instantaneous response—$g_{0}$, nonlinear contribution to creep deformation—$g_{1}$, nonlinear contribution to creep acceleration—$a_{\tau }$, and nonlinear contribution to recovery deformation—$g_{2}$. For linear viscoelastic response, the nonlinear parameters remain 1 and the given formulation is reduced to a well-established linear form.

The first irrecoverable expansion considers material flow describing progressive irreversible deformation even at infinitesimal external loads. Material flow is relevant for non-crosslinked molecular structures, such as thermoplastics (amorphous or semicrystalline), also known as the *rheodictic* class of polymers [9,33,40]. While amorphous polymers exhibit serious flow in the operating time–temperature scale, the flow of semi-crystalline polymers may be neglected until the material melts, as crystalline structure disables any relative movement of molecules. Irrecoverable material flow in the form of shear strain—$\gamma_{flow}\left(t\right)$ may be expressed as:

(3) $$\gamma_{flow}\left(t\right)=\phi_{flow}\int_{0}^{t}\tau (t)dt,$$

where $\phi_{flow}$ represents the flow term.

The second irrecoverable expansion considers viscoplastic behavior. Instead of the linear formulation as initially suggested by R. A. Schapery [7,8], various research works suggest using an exponential formulation to account for all viscoplastic processes (diminishing, progressive, damaging). To provide a comprehensive view of these processes, two models are considered. Perzyna’s model accommodates yield criterion, where parameters should be determined with a separate set of tests, i.e., tensile tests [34,35,36,37]. The model formulates viscoplastic strain rate—${\dot{\varepsilon }}_{vp}$ (or in shear ${\dot{\gamma }}_{vp}$) as:

(4) $${\dot{\varepsilon }}_{vp}=\frac{1}{\sqrt{3}}{\dot{\gamma }}_{vp}=\phi_{vp}\langle \Gamma (f)\rangle \frac{\partial f}{\partial \sigma_{ij}}=\phi_{vp}\left\langle {\left(\frac{f}{\sigma_{y}}\right)}^{n}\right\rangle \frac{\partial f}{\partial \sigma },$$

and f as a potential energy function using the Armstrong–Frederick model [36]:

(5) $$f=\sigma -\sigma_{y}=\frac{c}{k}\left(1-e^{-k\varepsilon_{vp}}\right),$$

where $C=\sqrt{3}\cdot \phi_{vp}$ presents viscoplastic flow term (for shear), n the process rate parameter, $\sigma_{y}$ yield stress, $\langle \Gamma (f)\rangle$ McCauly bracket (0 when f≤0, and ${\left(f/\sigma_{y}\right)}^{n}$ when f>0), and c, k as kinematic hardening parameters of f. On the other hand, Zappas and Crissman model does not accommodate the yield phenomenon; however, its parameters may be determined from creep recovery tests, the same tests as for the identification of nonlinear parameters [38,39]. In this case, viscoplastic strain—$\varepsilon_{vp}$ (or in shear $\gamma_{vp}$) is formulated as:

(6) $$\varepsilon_{vp}\left(t\right)=\frac{1}{\sqrt{3}}\gamma_{vp}\left(t\right)=\phi_{vp}{\left\{\int_{0}^{t}{\tau (t)}^{N}dt\right\}}^{n},$$

where $C=\sqrt{3}\cdot \phi_{vp}$ presents viscoplastic flow term (for shear), n the process rate parameter, and N stress scaling parameter. The parameter n portrays the nature of the viscoplastic process, which was in the later works of R. A. Schapery considered as 1 [7,8]. When n=1, material exhibits progressive or flow-like behavior, the formulation merges with Equation (3) forming some sort of nonlinear flow (similar to the one in [33]), which occurs at extreme environmental and loading conditions. Furthermore, the proposed formulation enables modeling also other viscoplastic processes observed in nature, such as diminishing viscoplastic processes that do not culminate in failure, where n<1, as well as damaging viscoplastic processes where n>1 that results in material rapture.

Both aforementioned models provide accurate predictions of viscoplastic deformations; however, their application primarily depends on the nature of material behavior, i.e., related to its structure (cross-linked vs. non-crosslinked) and whether it exhibits yield phenomena. While Perzyna’s model is suitable for cross-linked and composite materials with distanced yield phenomena, Zapass and Crissman’s model is appropriate for non-crosslinked materials where viscoplastic deformations may occur before yield, which will be considered in the present study.

The combined response of the material in the form of total shear strain—$\gamma_{total}\left(t\right)$, consists of (linear and) nonlinear viscoelastic component in combination with one or both plastic components portraying irrecoverable effect, such as material flow and viscoplastic deformation, plainly expressed as:

(7) $$\gamma_{total}\left(t\right)=\gamma_{nve}\left(t\right)+{\left\{\gamma_{flow}\left(t\right)+\gamma_{vp}\left(t\right)\right\}}_{p},$$

or extended to:

(8) $$\gamma_{total}\left(t\right)=g_{0}J_{0}\tau +g_{1}\int_{0}^{t}∆J\left(\psi -\psi^{\prime }\right)\frac{dd\left(g_{2}\tau \right)}{d\rho }d\rho +\phi_{flow}\int_{0}^{t}\tau (\rho )d\rho +C{\left\{\int_{0}^{t}{\tau (\rho )}^{N}d\rho \right\}}^{n}$$

Additionally, it has to be emphasized when addressing a complete constitutive description of material response that introduced irrecoverable components are present only in shear (change in geometry), and should be absent in bulk (change in volume), as they would lead to a singularity problem.

### 2.2. Reduction to Creep Recovery Data (Two-Step Loading Equation)

**Figure 1:**
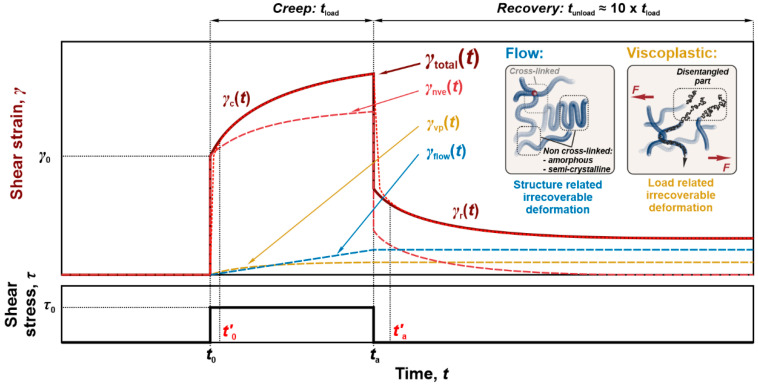
Schematic representation of the complete creep recovery response of the material consisting of (linear and) nonlinear viscoelastic components, as well as irrecoverable flow and viscoplastic components.

In the following section, $\gamma_{total}\left(t\right)$ will be reduced to the creep and recovery part of shear strain, i.e., $\gamma_{c}\left(t\right)$ and $\gamma_{r}\left(t\right)$, respectively, since those formulations enable identification of parameters within (linear and) nonlinear viscoelastic component, as well as in irrecoverable flow and viscoplastic components, Figure 1.

While the derivation of both viscoplastic components to creep recovery reduction is apparent, the thorough derivation of the nonlinear viscoelastic equation to creep recovery reduction may be found elsewhere [21]. Hance, the creep part of Equation (8) yields:

(9) $$\gamma_{c}\left(t\right)=\left[g_{0}J_{0}+g_{1}g_{2}\sum_{i=1}^{m}J_{i}(1-e^{-\;\frac{t}{a_{\tau }\lambda_{i}}})\right]\tau +\phi_{flow}t\tau +Ct^{n}\tau^{nN}.$$

whereas the recovery part of Equation (8) yields:

(10) $$\gamma_{r}\left(t\right)=g_{2}\left[\sum_{i=1}^{m}J_{i}\left(1-e^{-\;\frac{t_{a}}{a_{\tau }\lambda_{i}}}\right)e^{-\;\frac{t-t_{a}}{\lambda_{i}}}\right]\tau +\phi_{flow}t_{a}\tau +Ct_{a}^{n}\tau^{nN}.$$

Please note that the transient shear creep compliance—$∆J\left(t\right)$ portraying the linear viscoelastic response of the material, was expressed in the form of a Prony series as:

(11) $$∆J\left(t\right)=\sum_{i=1}^{n}J_{i}\left(1-e^{-\frac{t}{\lambda_{i}}}\right),$$

where $J_{i}$ and $\lambda_{i}$ represent the magnitude and time pairs of the so-called retardation time spectra $J_{i}\left(\lambda_{i}\right)$, respectively, portraying the contribution of the $i^{th}$ segment (molecular group) to overall viscoelastic response [38]. Moreover, $\gamma_{r}\left(t\right)$ may be further modified by introducing new time-independent but stress-dependent nonlinear retardation time spectra as [27]:

(12) $$A_{i}=J_{i}\left(1-e^{-\;\frac{t_{a}}{a_{\tau }\lambda_{i}}}\right)g_{2},$$

yielding:

(13) $$\gamma_{r}\left(t\right)=\left[\sum_{i=1}^{m}A_{i}e^{-\;\frac{t-t_{a}}{\lambda_{i}}}\right]\tau +\phi_{flow}t_{a}\tau +Ct_{a}^{n}\tau^{nN},$$

where determination of $A_{i}\left(\lambda_{i}\right)$ is essential to overcome certain ambiguities during parameter identification in the nonlinear viscoelastic domain.

### 2.3. Methodology for Determination of Nonlinear Viscoelastic–Viscoplastic Model Parameters

Throughout this section, the methodology to identify nonlinear Viscoelastic–Viscoplastic model parameters is explained. The presented methodology consists of 3 major steps. In the first step, the methodology analyzes linear viscoelastic behavior by determining $J_{i}\left(\lambda_{i}\right)$ from recovery data, while $J_{0}$ from the first point of creep data. In order to determine $\phi_{flow}$ creep tests should be performed, and the term should be extracted from the master curve. During the second step, the methodology analyzes nonlinear viscoelastic behavior, where the nonlinear parameters $g_{2}$ and $a_{\tau }$ were determined from recovery data, whereas $g_{0}$ were obtained from the fist and $g_{1}\;$ from the last point of the creep data. The last step of the methodology analyzes viscoplastic behavior from residual viscoplastic strains determined from the recovery part at $t_{unload}$, where only n was determined from creep.

Experimental and analytical methodology considers a sufficiently long recovery or unloading period, i.e., $t_{unload}=10\times t_{load}$ [33,41], during which (linear and) nonlinear viscoelastic material response settles, enabling accurate determination of residual strains from irrecoverable processes. Furthermore, since the real loading/unloading process is rate-dependent, $t_{0}^{\prime }$ and $t_{a}^{\prime }$ represent times at which theoretical and experimental creep and recovery strains converge (see Figure 1). During creep the load was applied in 0.1 s, hence $t_{0}^{\prime }$ was taken at 1 s (10× of the loading process), while during recovery, the load was released in 1 s to mitigate the ringing effect, $t_{a}^{\prime }$ was determined at 10 s (10× of the unloading process). Consequently, the evaluations during recovery consider the timeframe between $t_{a}^{\prime }$ and $t_{unload}$, while during creep, between $t_{0}^{\prime }$ and $t_{a}$, assuming that irrecoverable processes have a negligible effect at $t_{0}^{\prime }$.

#### 2.3.1. Linear Viscoelastic Behavior

Methodology starts by evaluating the linear viscoelastic behavior of a material as nonlinear parameters remain equal to 1. In order to avoid an instantaneous response, which is difficult to measure, the recovery part is considered first. With the aim of analyzing the pure linear viscoelastic response by eliminating an irrecoverable effect, differential values of the recovery data and model from Equation (10) were used, as shown in the following expression:

(14) $$\gamma_{r}^{exp}\left(t\right)-\gamma_{r}^{exp}\left(t_{unload}\right)=\left[\sum_{i=1}^{m}J_{i}\left(1-e^{-\;\frac{t_{a}}{\lambda_{i}}}\right)e^{-\;\frac{t-t_{a}}{\lambda_{i}}}-\sum_{i=1}^{m}J_{i}\left(1-e^{-\;\frac{t_{a}}{\lambda_{i}}}\right)e^{-\;\frac{t_{unload}-t_{a}}{\lambda_{i}}}\right]\tau$$

where the magnitude and time pairs of the retardation time spectra $J_{i}\left(\lambda_{i}\right)$ can be approximated from Equation (14) by using any established least square optimization algorithms (Levenberg–Marquardt, trust region reflective, etc.) or any existing algorithms for spectral analysis (widowing, edge preserving, etc.) [42,43,44]. The number and positions of $\lambda_{i}$ should follow recommendations from [9].

Finally, a good approximation of $J_{0}$ can be obtained from the first point of creep data at $t_{0}^{\prime }$, by accounting the linear viscoelastic part while assuming a negligible contribution of any irrecoverable effects. Such assumptions are valid, since their contribution to $J_{0}$ usually ranges from $10^{-6}$ to $10^{-9}1/MPa$ which is a thousand times smaller, simplifying Equation (9) into:

(15) $$\gamma_{c}^{exp}\left(t_{0}^{\prime }\right)=\left[J_{0}+\sum_{i=1}^{m}J_{i}(1-e^{-\;\frac{t_{0}^{\prime }}{\lambda_{i}}})\right]\tau$$

If a material exhibits notable flow, $\phi_{flow}$ should be identified from separate experiments, suggesting creep or relaxation tests either in the time or frequency domain at elevated temperatures coupled with a time–temperature superposition, for which a detailed explanation of experimental and analytical methodology can be found in various literature [1,9,40].

#### 2.3.2. Nonlinear Viscoelastic Behavior

As suggested by R. A. Schapery, to avoid instantaneous contribution, which is difficult to identify, the methodology for evaluating nonlinear viscoelastic behavior of material should be initiated from recovery [21]. Similarly as before, a pure nonlinear viscoelastic response by eliminating irrecoverable effect can be determined from differential values of recovery data and model from Equation (13) as shown in the following expression:

(16) $$\gamma_{r}^{exp}\left(t\right)-\gamma_{r}^{exp}\left(t_{unload}\right)=\left[\sum_{i=1}^{m}A_{i}e^{-\;\frac{t-t_{a}}{\lambda_{i}}}-\sum_{i=1}^{m}A_{i}e^{-\;\frac{t_{unload}-t_{a}}{\lambda_{i}}}\right]\tau .$$

where the magnitude and time pairs of the nonlinear retardation time spectra $A_{i}\left(\lambda_{i}\right)$ can be approximated from Equation (16) applying any previously mentioned minimization procedure. Using the same idea, $a_{\tau }$ can be approximated from Equation (10):

(17) $$\gamma_{r}^{exp}\left(t\right)-\gamma_{r}^{exp}\left(t_{unload}\right)={\left\{\frac{\sum_{i=1}^{m}A_{i}}{\sum_{i=1}^{m}J_{i}\left(1-e^{-t_{a}/\left(a_{\tau }\lambda_{i}\right)\;}\right)}\right\}}_{g_{2}}\left[\sum_{i=1}^{m}J_{i}\left(1-e^{-\;\frac{t_{a}}{a_{\tau }\lambda_{i}}}\right)e^{-\;\frac{t-t_{a}}{\lambda_{i}}}-\sum_{i=1}^{m}J_{i}\left(1-e^{-\frac{t_{a}}{a_{\tau }\lambda_{i}}}\right)e^{-\;\frac{t_{unload}-t_{a}}{\lambda_{i}}}\right]\tau .$$

where $g_{2}$ is derived from the sum of Equation (12) and can be calculated as soon as the $a_{\tau }$ is known.

Since nonlinear viscoelastic, flow and viscoplastic mechanisms concurrently enroll during creep, nonlinear parameters $g_{0}$ and $g_{1}$ can be determined at the points where the magnitude of irrecoverable processes is either known or can be neglected. A good approximation of $g_{0}$ can be obtained from the first point of creep data at $t_{0}^{\prime }$ by accounting nonlinear viscoelastic part while considering same assumptions as before, simplifying Equation (9) to:

(18) $$\gamma_{c}^{exp}\left(t_{0}^{\prime }\right)=\left[g_{0}J_{0}+{\left\{\frac{g_{0}J_{0}\tau -\phi_{flow}t_{a}\tau -\gamma_{vp}(t_{a})}{{\tau g}_{2}\sum_{i=1}^{m}J_{i}\left(1-e^{-t_{a}/\left(a_{\tau }\lambda_{i}\right)\;}\right)}\right\}}_{g_{1}}g_{2}\sum_{i=1}^{m}J_{i}\left(1-e^{-\;\frac{t_{0}^{\prime }}{a_{\tau }\lambda_{i}}}\right)\right]\tau$$

where $g_{1}$ is derived from Equation (9) at $t_{a}$, which can be calculated once $g_{0}$ is available. Be aware that viscoplastic residuals are known from the recovery and will be thoroughly examined in continuation.

#### 2.3.3. Viscoplastic Behavior

The methodology to analyze the viscoplastic behavior of the material begins with identification of residual viscoplastic strains—$\gamma_{vp}(t_{a})$ from Equation (10), determined as a difference between recovery data and model at $t_{unload}$ (the time at which material viscoelastic response settles), yielding:

(19) $$\gamma_{vp}\left(t_{a}\right)=\gamma_{r}^{exp}\left(t_{unload}\right)-g_{2}\left[\sum_{i=1}^{m}J_{i}\left(1-e^{-\;\frac{t}{a_{\tau }\lambda_{i}}}\right)e^{-\;\frac{t_{unload}-t_{a}}{\lambda_{i}}}\right]\tau -\phi_{flow}t_{a}\tau$$

The analysis is performed for each applied τ and values correspond to viscoplastic model at $t_{a}$ as:

(20) $$\gamma_{vp}\left(t_{a}\right)=Ct_{a}^{n}\tau^{nN}$$

Viscoplastic parameters can be identified by using logarithmic trick on Equation (20) which linearizes exponential function in the following expression:

(21) $$\log\gamma_{vp}\left(t_{a}\right)=nN\log\tau +\log Ct_{a}^{n},$$

where product n·N represents the slope, while logarithmic value of $K=Ct_{a}^{n}$ is the intersection of given linear function. Average value of n can be approximated from viscoplastic strain—$\gamma_{vp}\left(t\right)$ using Equation (9) determined as a difference between the creep data for each τ, yielding:

(22) $$\gamma_{vp}\left(t\right)=\gamma_{c}^{exp}\left(t\right)-\left[g_{0}J_{0}+g_{1}g_{2}\sum_{i=1}^{m}J_{i}\left(1-e^{-\;\frac{t}{a_{\tau }\lambda_{i}}}\right)\right]\tau -\phi_{flow}t\tau ={\left\{\frac{K}{t_{a}^{n}}\right\}}_{C}t^{n}\tau^{nN}.$$

Once n is identified, the parameter N can be calculated from the slope, and the parameter C from the intersection of Equation (21), respectively.

## 3. Experimental Part

### 3.1. Materials and Sample Preparation

The study of nonlinear viscoelastic–viscoplastic behavior was conducted on two versatile and well-established thermoplastic polymers with different morphological structures: amorphous acrylonitrile butadiene styrene—ABS (P3H-AT, Elix polymers, La Canonja, Spain) and semi-crystalline polyoxymethylene—POM (Hostaform 27021, Celanese, Irving, TX, USA). The selection of the materials was predominantly based on the different behaviors that those structures portray. Due to the nature of chemical bonds (non-crosslinked system) they are both considered as rheodictic class of polymers (exhibiting irrecoverable deformations); however, their morphological differences (disordered vs. ordered chain structure) enable flow at substantially different time–temperature scales. While amorphous ABS exhibits profound flow within the considered temperature range, the flow of semi-crystalline POM may be neglected until its melting.

Samples of the investigated materials were prepared using a three-step manufacturing procedure: drying, melt mixing and injection molding. In the first step, granules were dried in a laboratory oven (SP105C, Kambič d.o.o., Semič, Slovenia) to remove diffused moisture and therefore avoid bubbling during mixing and molding procedures. During the second step, granules were mixed in a mini twin-screw extruder (MC15HT, Xplore Instruments BV, Sittard, The Netherlands) to remove trapped air and to obtain homogeneous melt. Within the third step, melt was transported to a mini-injection molding machine (IM12, Xplore Instruments BV, Sittard, The Netherlands) to prepare cylindrically shaped samples with diameter of d = 3 mm and length of l = 50 mm. In the process the injection pressures were selected to enable outgassing while minimizing residual thermo-mechanical stresses after the molding process. The conditions during the three-step manufacturing procedure of the samples are summarized in Table 1.

### 3.2. Thermal Conditioning and Evaluation of Thermodynamic Equilibrium

Thermal conditioning eliminates (or at least substantially reduces) residual thermomechanical stresses that persist within the samples after pressure/shear-intensive manufacturing processes. If it is not properly mitigated or thermodynamic equilibrium is not reached, they lead to a physical aging process (a change in physical properties over time without any external excitation) resulting in significant errors when evaluating time-dependent behavior of polymers [3,45]. To analyze the thermal conditioning and corresponding thermodynamic state of the prepared samples, the (TC-TD) procedure was divided into three steps [33,46]: (i.) determination of a glass transition temperature, (ii.) thermal conditioning, and iii) evaluation of thermodynamic equilibrium.

Within the first step, thermal analysis was performed on investigated materials to determine the glass transition temperature, $T_{g}$, a temperature where relaxation or stress release processes accelerate. Measurements were carried out on differential scanning calorimeter, DSC (Q2500, TA Instruments, USA), in a temperature range from 0 °C to 250 °C for amorphous ABS, and from −90 °C to 250 °C for semi-crystalline POM. All tests were performed according to the standard ISO 11,357 [47] with heating and cooling rates of 10 °C/min under a nitrogen atmosphere to prevent material oxidation. During the second step, the samples of the selected materials under investigation were conditioned in a laboratory oven (SP105C, Kambič, Slovenia) enclosed in a glass chamber under a nitrogen atmosphere and conditioned at the temperature of $T_{con}\approx 100\;{}^{\circ}C$ (for amorphous ABS); $T_{con}$ also corresponds to an onset value of $T_{g}$ preserving the geometry of ABS samples throughout the conditioning process. Samples were conditioned for $t_{con,1.5}\approx 1.5\;h$, $t_{con,3}\approx 3\;h$, and $t_{con,6}\approx 6\;h$, and then slowly cooled to room temperature with a cooling rate of ${\dot{T}}_{con}\approx 0.1\;{}^{\circ}C/min\;$to mitigate any aging effects. In the third step, dynamic thermomechanical analysis, DTMA, was performed on the samples of studied materials to evaluate thermodynamic equilibrium by observing changes in viscoelastic properties (storage $G^{\prime }$ and loss $G^{\prime \prime }$ moduli) resulting from the above-mentioned conditioning procedures. Measurements were carried out on advanced modular rheological system (MCR702, Anton Paar, Austria) coupled with a CTD180 temperature chamber and using solid circular fixtures or SCF sensors. All tests were performed according to standard ISO 6721 [48] in a shear mode of loading applying stress of 1 MPa (within linear viscoelastic domain) at a frequency of 1 Hz, in a temperature range from 0 °C to 110 °C for amorphous ABS, and from 0 °C to 100 °C for semi-crystalline POM, with a heating rate of 3 °C/min. The DTMA results present the average value of three repetitions (each on fresh sample), and the error corresponds to maximum deviation from the average value.

**Figure 2:**
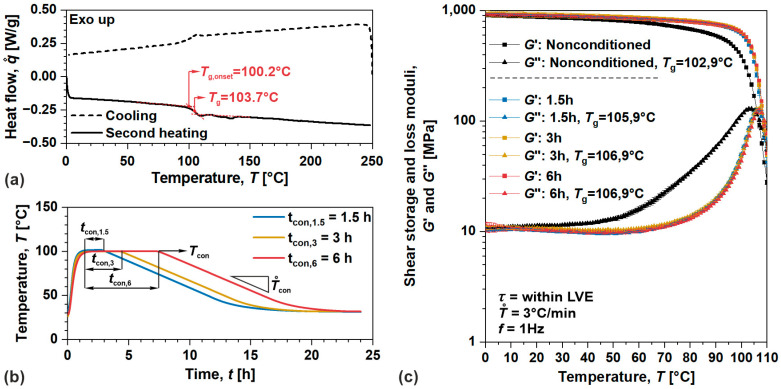
(**a**) Thermal analysis of amorphous ABS, (**b**) recorded temperature profiles during thermal conditioning of the samples, and (**c**) DTMA analysis of nonconditioned and conditioned samples.

The results of the complete TC-TD procedure for amorphous ABS samples are shown in Figure 2. From thermal analysis (DSC thermograms), the glass transition temperature was determined to be $T_{g}\approx 103.7\;{}^{\circ}C$ with the onset value of $T_{g,onset}\approx 100.2\;{}^{\circ}C$, Figure 2a. Samples were then conditioned as portrayed by recorded temperature profiles, Figure 2b. The DTMA results show profound differences in viscoelastic properties as well as $T_{g}$ between nonconditioned and conditioned samples. During the manufacturing of amorphous ABS samples, molecules “froze” upon solidification significantly below $T_{g}$. Therefore, nonconditioned samples in comparison to the conditioned ones exhibited expended free volume/enthalpy (available volume or potential energy for molecular rearrangement) identified from decreased $G^{\prime }$ (corresponds to stored/recoverable energy), increased $G^{\prime \prime }$ (corresponds to dissipated/consumed energy) and lower $T_{g}$ (elevated segmental mobility) [49,50], indicating sever residual thermomechanical stresses within the samples, Figure 2c. Nevertheless, negligible changes in viscoelastic properties (within experimental error) were observed for the samples conditioned at $t_{con,3}$ and $t_{con,6}$, implying predominant elimination of those stresses and achievement of thermodynamic equilibrium.

**Figure 3:**
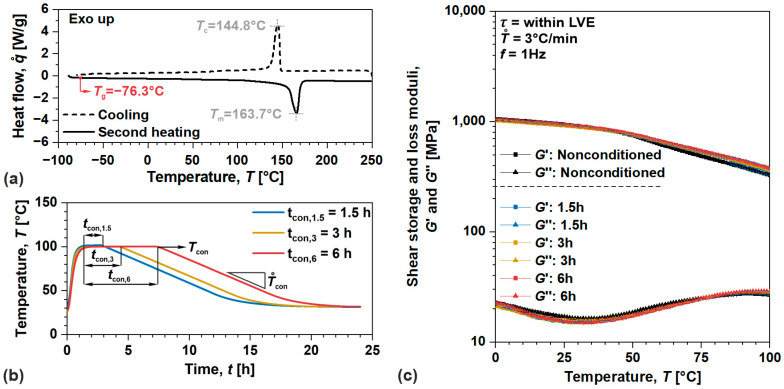
(**a**) Thermal analysis of semi-crystalline POM, (**b**) recorded temperature profiles during thermal conditioning of the samples, and (**c**) DTMA analysis of nonconditioned and conditioned samples.

Figure 3 shows the results of complete TC-TD procedure for semi-crystalline POM samples. From thermal analysis (DSC thermograms), glass transition was determined at temperature of $T_{g}\approx -76.3\;{}^{\circ}C$, Figure 3a. Samples were conditioned after heating as portrayed by the recorded temperature profiles, Figure 3b. The DTMA results show minor differences in viscoelastic moduli between the nonconditioned and conditioned sample. In this case, during the manufacturing of semi-crystalline POM samples, the molecules “froze” upon solidification significantly above $T_{g}$. Hence, nonconditioned samples in comparison to the conditioned ones exhibit marginal differences in free volume as identified from minor changes in viscoelastic moduli, indicating some thermomechanical stresses still remained within the samples, Figure 3c. However, negligible changes in viscoelastic properties (within experimental error) were observed for all conditioning procedures, indicating complete elimination of residual stresses within the samples, and achievement of thermodynamic equilibrium. As portrayed above, several thermal conditioning procedures allow the samples of investigated materials to achieve a thermodynamic equilibrium. However, to minimize the risk of aging effects, $t_{con,6}$ was selected for further characterization of the nonlinear viscoelastic–viscoplastic behavior of the polymers studied.

### 3.3. Creep Recovery Tests for Nonlinear Viscoelastic–Viscoplastic Analysis

For nonlinear viscoelastic–viscoplastic analysis, creep recovery tests were carried out on an advanced modular rheological system (MCR702, Anton Paar, Graz, Austria) coupled with a CTD180 temperature chamber and a SCF sensor geometry. Measurements were performed at constant temperature of $T_{exp}=70\;{}^{\circ}C$ (maximum operating temperature) in shear stress mode of loading by applying shear stresses (i) τ=0.1, 1 and 2.5 MPa to determine the parameters in linear viscoelastic domain; and (ii) τ= 5, 7.5, 10, 12.5, 15 MPa (~70% strength, calculated from UTS data) to determine the parameters in nonlinear viscoelastic domain. During the creep phase, the samples were loaded for 1800 s (0.5 h), while during the recovery phase they were unloaded for 18,000 s (5 h), which was sufficiently long to obtain the complete recovery, i.e., 10× longer than the creep phase as reported in several studies [33,41]. Please note that the same experimental conditions were applied on the samples of both investigated materials, i.e., amorphous ABS and semi-crystalline POM.

Moreover, due to the inherent rheodictic nature of amorphous ABS, a flow of the material was determined from long-term behavior portrayed by material function. In this particular case, creep measurements were performed in a shear mode of loading by applying τ of 1 MPa (within linear viscoelastic domain) for 1000 s in a temperature range from 30 to 100 °C (with a step of 10 °C bellow 80 °C and a step of 5 °C above 80 °C). With the use of time-temperature superposition tTs principle, isothermal segments of shear creep compliance were then shifted (horizontal and vertical shift) in order to construct master curve or material function at the reference temperature of $T_{ref}=70\;{}^{\circ}C$ (the same temperature as used in creep recovery tests) representing the behavior of the material through a longer period of time.

The results of creep recovery tests and creep tests present the average value of three repetitions (each on fresh sample), and the error corresponds to maximum deviation from the average value.

## 4. Results and Discussion

### 4.1. Linear Viscoelastic Behavior of Polymers

In this section, linear viscoelastic behavior of investigated materials is addressed. Multiscale analysis was performed (following methodology discussed in Section 2.3) to identify linear viscoelastic model parameters, consisting of elastic component as instantaneous shear creep compliance—$J_{0}$, viscoelastic component or shear retardation time spectra $J_{i}(\lambda_{i})$ for semi-crystalline POM, and additionally flow component as the material flow or flow term $\phi_{flow}$ for amorphous ABS.

**Figure 4:**
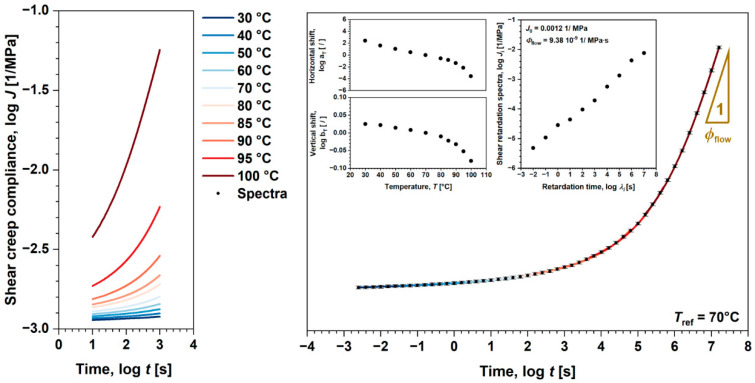
Segments and master curve of shear creep compliance for amorphous ABS, including horizontal and vertical shifts, as well as shear retardation time spectra.

First, we have evaluated linear viscoelastic behavior and model parameters for amorphous ABS. Since the material at 70 °C does not exhibit serious flow, the evaluation from recovery data may lead to significant overestimation, which can incorporate other, although small viscoplastic effects. Hence, the flow component was determined from the results of creep tests. Resulting isothermal segments of shear creep compliance J(t) were shifted according to tTs principle in order to construct a master curve (material function) at the reference temperature of 70 °C, Figure 4. It is important to note that horizontal—$\log a_{T}$ and vertical $\log b_{T}$ shifts were employed, where $b_{T}$ is defined as reduced variable of $J_{red}\left(t\right)=J(t)/b_{T}$, following its physical interpretation given by J. D. Ferry in [40]. As a result, $\phi_{flow}$ may be determined, either from the slope of the constructed master curve (represented by the segment measured at the highest temperature where the material exhibits a profound flow) or from modeling of such behavior (by spectral analysis utilizing least squares optimization algorithm) as articulated in the work of N. W. Tschoegl [9]. Anyhow, both approaches were used and provided a similar value of $\phi_{flow}=9.38\times 10^{-9}\;1/MPa\cdot s$, with negligible differences between the two, i.e., <1%.

**Figure 5:**
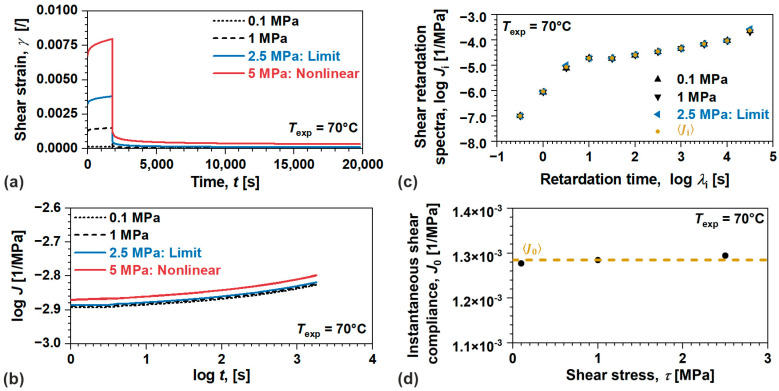
(**a**) Shear strain during creep recovery test, (**b**) shear creep compliance, (**c**) instantaneous shear creep compliance (elastic component), and (**d**) shear retardation spectra (viscoelastic component) in linear viscoelastic domain for amorphous ABS.

Elastic and viscoelastic components of linear viscoelastic model for amorphous ABS were determined from the results of creep recovery tests performed at 70 °C, Figure 5. For convenience, shear strain $\gamma \left(t\right)$ at 5 MPa is also displayed to demonstrate nonlinear viscoelastic behavior, Figure 5a. Such behavior may be observed as a collapse of proportionality and additivity between the load and response, the two main conditions based on which linear viscoelastic theory is postulated [9,10], portrayed by the change in J(t) during creep, Figure 5b. Based on the results, the shear stress limit of linear viscoelasticity was determined to be $\tau_{LVE}=2.5\;MPa$. To avoid instantaneous response as it is difficult to measure [21,33], viscoelastic component portrayed by $J_{i}(\lambda_{i})$ was determined through spectral analysis using least squares optimization algorithm over differential values of $\gamma \left(t\right)$ during the recovery process. This procedure determines pure viscoelastic response by eliminating (as well as identifying) flow and viscoplastic residual strains. Furthermore, the average value was determined as $\langle J_{i}\rangle (\lambda_{i})$ by considering all loading conditions in linear domain, Figure 5c.

Please note that the number and positions of the retardation times $\lambda_{i}$ were predetermined using recommendations from [9]. Finaly, good approximation of elastic component given as $J_{0}$ was determined from the first point of measured data while extracting viscoelastic part at that time (1 s), and assuming insignificant contribution of irrecoverable processes (flow and viscoplastic). The average value was determined as $\langle J_{0}\rangle =1.28\times 10^{-3}\;1/MPa$ by considering all loading conditions in the linear viscoelastic domain, Figure 5d.

**Figure 6:**
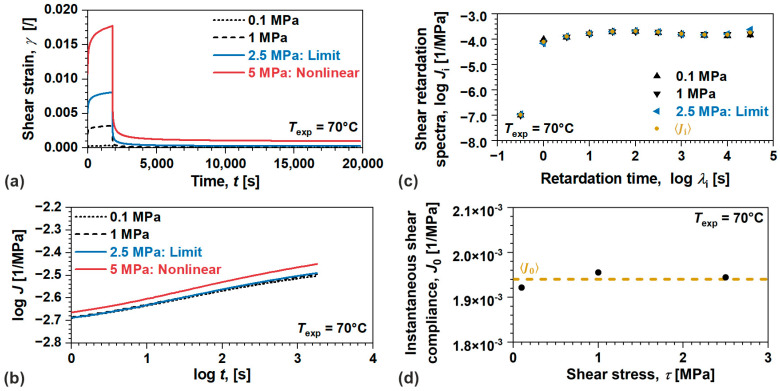
(**a**) Shear strain during creep recovery test, (**b**) shear creep compliance, (**c**) instantaneous shear creep compliance (elastic component), and (**d**) shear retardation spectra (viscoelastic component) in linear viscoelastic domain for semi-crystalline POM.

Now, we will analyze the linear viscoelastic behavior and model parameters for semi-crystalline POM, Figure 6. Since the flow of this material may be neglected within considered time–temperature scale, elastic and viscoelastic components were determined from the results of creep recovery tests performed at 70 °C, Figure 6a. For convenience, $\gamma \left(t\right)$ at 5 MPa is also displayed to illustrate nonlinear viscoelastic behavior. By observing the change in J(t) during creep, the limit was determined to be $\tau_{LVE}=2.5\;MPa$ with the collapse of proportionality and additivity, Figure 6b. Furthermore, the average value of viscoelastic component was evaluated as $\langle J_{i}\rangle (\lambda_{i})$, and ultimately, the average value of instantaneous response was identified as $\langle J_{0}\rangle =1.94\times 10^{-3}\;1/MPa$ by considering all loading conditions in the linear viscoelastic domain, Figure 6c,d, respectively.

### 4.2. Nonlinear Viscoelastic Behavior of Polymers

Within this section, the nonlinear viscoelastic behavior of the studied materials is discussed. Multi-scale analysis was carried out (following methodology discussed in Section 2.3) to determine Schapery’s nonlinear viscoelastic model parameter. Those parameters provide information on nonlinear (stress-dependent) contributions to linear viscoelastic responses by reflecting third- and higher-order dependencies of Gibb’s free energy (potential energy for molecular reconfiguration) [21]; namely, nonlinear contribution to instantaneous response $g_{0}$, nonlinear contribution to creep deformation $g_{1}$, nonlinear contribution to creep acceleration $a_{\tau }$, and nonlinear contribution to recovery deformation $g_{2}$ [33].

**Figure 7:**
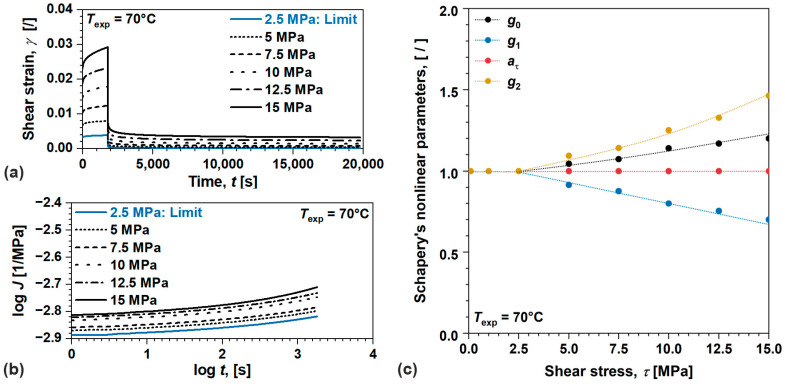
(**a**) Shear strain during creep recovery test, (**b**) shear creep compliance, (**c**) Schapery’s parameters in nonlinear viscoelastic domain for amorphous ABS.

We started by examining nonlinear viscoelastic behavior and model parameters for amorphous ABS, Figure 7. Those parameters were identified from the results of creep recovery tests performed at 70 °C, Figure 7a. For convenience, shear strain $\gamma \left(t\right)$ at 2.5 MPa is also shown to indicate the limit of linear viscoelastic behavior. The collapse of proportionality and additivity may be observed by the changes in shear creep compliance—J(t) for each applied shear stress τ, portraying consistent nonlinear viscoelastic behavior throughout the considered loading conditions, Figure 7b [6,11,21]. Similarly as in the linear domain, to avoid instantaneous contribution, which is difficult to measure, $a_{\tau }$ and $g_{2}$ were determined first from the recovery part of the data [21,33]. Both parameters were determined for each applied load using least squares optimization algorithm over differential values of $\gamma \left(t\right)$ during recovery. This procedure enables the determination of pure nonlinear viscoelastic response by eliminating (as well as identifying) flow and viscoplastic residual strains. Finally, good approximates of $g_{1}$ and $g_{0}$ were determined from $\gamma \left(t\right)$ during creep using similar minimization methods. Since nonlinear viscoelastic, flow and viscoplastic mechanisms simultaneously occur during creep, both parameters were determined at points where the magnitude of irrecoverable processes (flow and viscoplastic) is either known or can be neglected.

Therefore, parameter $g_{1}$ was determined at the end of creep process, i.e., 1800 s where residual strains were identified from the recovery, while $g_{0}$ was determined from as the start of creep process, i.e., 1 s, assuming insignificant contribution of irrecoverable effects. Results show various dependencies of Schapery’s nonlinear parameters with τ, i.e., $g_{0}(\tau )$—linear, $g_{1}(\tau )$—linear, $a_{\tau }(\tau )$—constant, and $g_{2}(\tau )$—quadratic, Figure 7c. As reported in various research works they depend on polymer, temperature, type of loading, etc. [24,25,26,27,28,29,30]. Most surprising is the decrease in $g_{1}\left(\tau \right)$ although $a_{\tau }(\tau )$ remains 1, which was historically predicted by R.A. Schapery [21] for a special class of polymers, presumably related to the concurrent flow and viscoplastic processes during creep. Nevertheless, the product $g_{1}g_{2}$ which drives the magnitude of creep process was found to be increasing with increasing τ.

**Figure 8:**
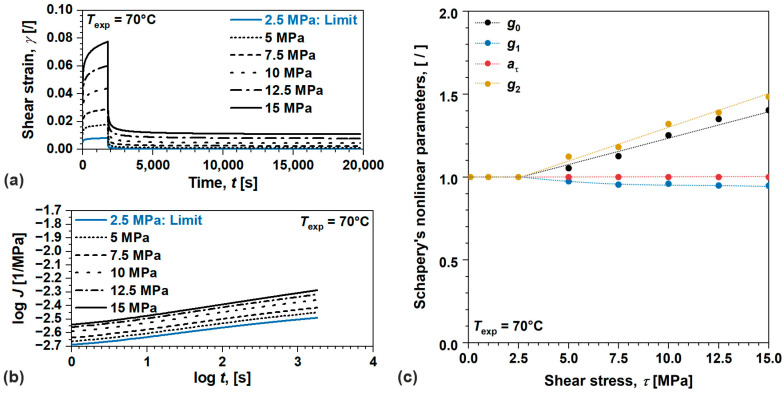
(**a**) Shear strain during creep recovery test, (**b**) shear creep compliance, (**c**) Schapery’s parameters in nonlinear viscoelastic domain for semi-crystalline POM.

Next, we will evaluate nonlinear viscoelastic behavior and model parameters for semi-crystalline POM, Figure 8. The parameters were identified from the results of creep recovery tests performed at 70 °C, Figure 8a. For convenience, shear strain—$\gamma \left(t\right)$ at 2.5 MPa is also displayed to indicate limit of linear viscoelastic behavior. The material exhibited consistent nonlinear viscoelastic behavior over τ range observed by the changes in J(t), Figure 8b. Similarly as before, nonlinear parameters were determined for each τ using least squares optimization algorithm. First $a_{\tau }$ and $g_{2}$ were determined from $\gamma \left(t\right)$ during recovery, avoiding the difficulties related to measurements of instantaneous contribution. Good approximates of $g_{1}$ and $g_{0}$ were determined from $\gamma \left(t\right)$ during creep, under the same assumption as previously discussed. The results show different stress-dependent parameters, i.e., $g_{0}(\tau )$—linear, $g_{1}(\tau )$—quadratic, $a_{\tau }(\tau )$—constant, and $g_{2}(\tau )$—linear, as earlier observed in amorphous ABS, originating from morphological differences, thermodynamic state in relation to the glass transition, etc., Figure 8c. Furthermore, $g_{1}\left(\tau \right)$ also decreases although $a_{\tau }(\tau )$ remain 1, apparently related to the concurrent viscoplastic process occurring during creep; however, the decrease is ~27% smaller compared to amorphous ABS, since semi-crystalline POM does not exhibit flow within the considered time–temperature scale. Similarly as before, $g_{1}\left(\tau \right)$ decreases although $a_{\tau }(\tau )$ remains 1, presumably related to the concurrent viscoplastic processes during creep; however, the product $g_{1}g_{2}$ which drives the magnitude of creep process was found to be increasing with increasing τ.

### 4.3. Viscoplastic Behavior of Polymers

Throughout this section, the viscoplastic behavior of the investigated polymers will be examined. Analysis was conducted (following methodology discussed in Section 2.3) to evaluate viscoplastic model parameters, including viscoplastic flow term—C, stress scaling parameter—N, and process rate parameter—n [38,39]. Since the measurements were conducted in shear, which represents a natural loading condition for identifying irrecoverable processes (flow and viscoplastic), we were able to identify viscoplastic residual strains even within linear viscoelastic domain. Those viscoplastic strains, which presence was generally neglected as the magnitudes were practically insignificant [21], are not associated with yield phenomena (associated with progressive/damaging mechanisms) as commonly referred to in the literature, but rather explain the material’s inability to fully recover.

**Figure 9:**
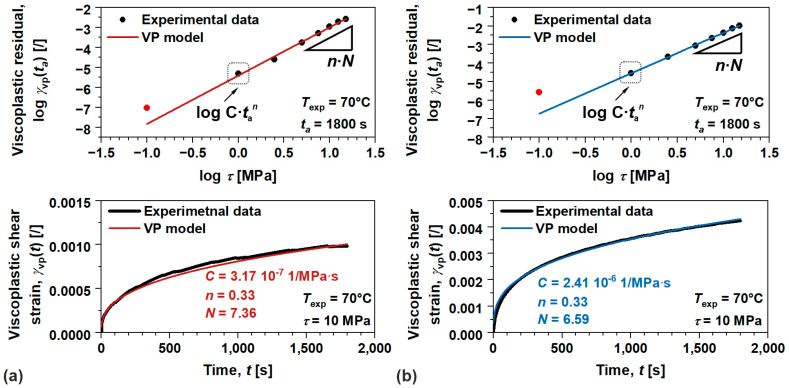
Comparison between experimental data and viscoplastic model for viscoplastic residuals as well as viscoplastic strain during creep on (**a**) amorphous ABS, and (**b**) semi-crystalline POM.

Anyway, viscoplastic parameters were identified from the results of creep recovery tests performed at 70 °C in linear and nonlinear viscoelastic domain. From recovery part of the data, a residual viscoplastic strain $\gamma_{vp}(t_{a})$ was identified (for amorphous ABS the effect of flow was extracted), Figure 9.

Apparently, $\gamma_{vp}\left(t_{a}\right)$ exponentially increases with shear stress τ, which in double-logarithmic scale/values, such dependency, translates into linear form, where n·N product presents the slope and logarithmic value of $Ct_{a}^{n}$ intersection. Similar behavior was observed with viscoplastic strain rates [51,52], while other research works show the same behavior with increasing $t_{a}$, where the slope determines the parameter n; however, additional set of experiments were needed [38,39]. In our case, the average value of n was determined for each loading condition using least square optimization procedure over $\gamma_{vp}(t)$ during creep. While N was then trivially calculated from the slope, the C was calculated from the intersection. Such a procedure allows identification of model parameters with single set of experiments. Anyway, the results show excellent agreement with the viscoplastic model with experimental data throughout the complete τ range over $\gamma_{vp.r}(t_{a})$ and time over $\gamma_{vp}(t)$ (shown on example for 10 MPa, Figure 9). It should be noted that 0.1 MPa (marked as a red dot in Figure 9) was not considered in the viscoplastic analysis, presumably due to limiting measuring capabilities of the device.

### 4.4. Evaluation of Nonlinear Viscoelastic–Viscoplastic Model in Linear and Nonlinear Domain

In the following section, the nonlinear viscoelastic–viscoplastic model performance of the studied polymers will be evaluated by comparing the predicted values of shear strain $\gamma \left(t\right)$ with those obtained from the creep recovery tests. For the convenience of such analysis, the model parameters for both considered materials are given in Table 2.

**Figure 10:**
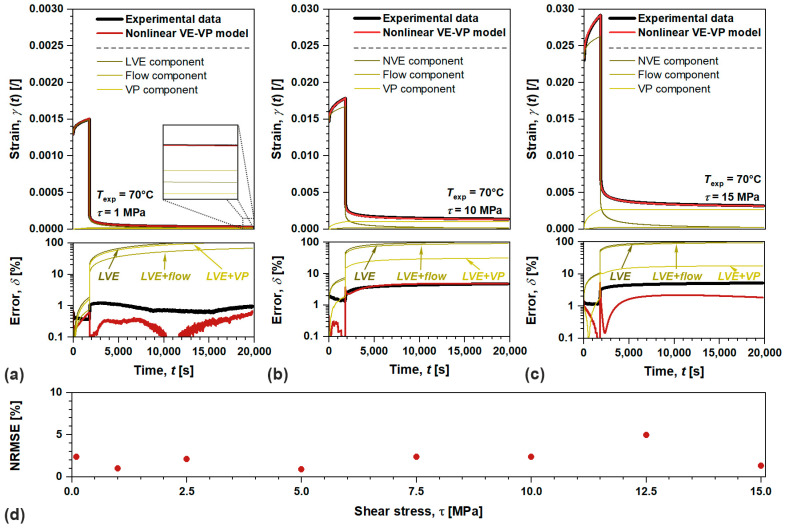
Showcasing nonlinear VE-VP model performance on creep recovery data with residuals at (**a**) 1 MPa (linear domain), (**b**) 10 MPa (nonlinear domain), and (**c**) 15 MPa (nonlinear domain), along with its overall accuracy portrayed by (**d**) normalized root mean square error for amorphous ABS.

In the first step we will analyze nonlinear viscoelastic–viscoplastic model performance for amorphous ABS, Figure 10. There are various parameters to evaluate such performance, among them, normalized root mean square error NRMSE (in %) more comprehensively quantify the goodness of the fit, which summarizes model accuracy and its predictive capability [53]. Evaluation procedure was carried out for all loading conditions in linear and nonlinear viscoelastic domain as showcased by Figure 10a–c. Since amorphous ABS exhibits notable flow in considered time–temperature scale, the related component governs the irrecoverable deformation in linear domain, while viscoplastic one governs those in nonlinear domain (the impact of flow diminishes). Anyway, the results indicate excellent agreement between the proposed model and the experimental data as NRMSE was determined <5% for each applied shear stress τ, supporting the methodology and introduced expansions of irrecoverable components, i.e., flow and viscoplastic, to Schapery’s nonlinear viscoelastic model, Figure 10d. Additionally, Figure 10 also includes relative experimental and analytical error δ. An abrupt increase in δ occurs during the loading/unloading process, as the samples undergo sudden geometrical change resulting from the application/release of τ. Furthermore, ablation tests portray the significance of introduced flow and viscoplastic components into nonlinear VE model as the error profoundly increases ranging from ~17 to ~110% over applied τ, if one of them is excluded from the evaluation.

**Figure 11:**
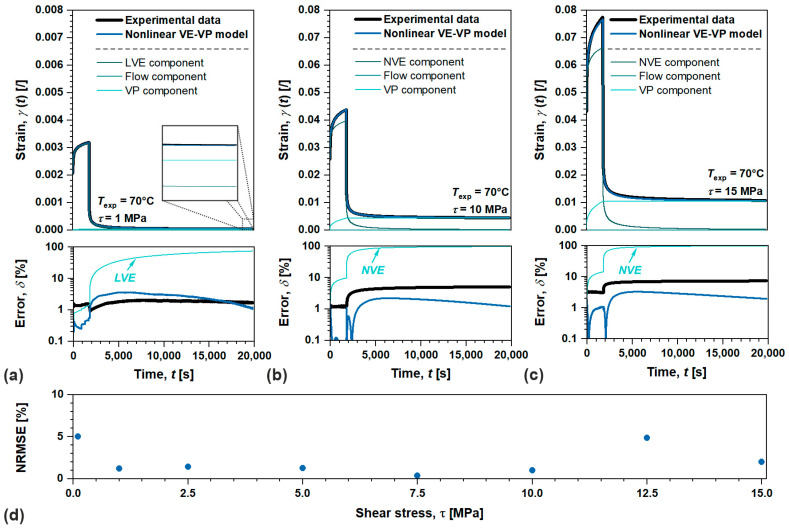
Showcasing nonlinear VE-VP model performance on creep recovery data with residuals at (**a**) 1 MPa (linear domain), (**b**) 10 MPa (nonlinear domain), and (**c**) 15 MPa (nonlinear domain), along with its overall accuracy portrayed by (**d**) normalized root mean square error for semi-crystalline POM.

Similarly as before, we examined the nonlinear viscoelastic–viscoplastic model performance also for semi-crystalline POM, Figure 11. Analysis was performed for all loading conditions in the linear and nonlinear viscoelastic domains as showcased by Figure 11a to 11c (relative experimental and analytical errors are also included).

Since semi-crystalline POM does not exhibit flow in the observed time–temperature scale, the irrecoverable deformations are solely governed by the viscoplastic component in the linear and nonlinear domains. The results show excellent agreement between the proposed model and the experimental data with NRMSE <5% for each τ, confirming the introduced expansions to Schapery’s nonlinear model. Furthermore, ablation tests were performed to illustrate the importance of introduced viscoplastic components in to nonlinear VE model, since the error profoundly increases ranging from ~74 to ~10% over applied τ, if it is excluded from analysis.

Based on the obtained results, the nonlinear viscoelastic–viscoplastic model allows the prediction of time-dependent behavior with exceptional accuracy, regardless of the morphological nature and property of the material. Therefore, the proposed analytical and experimental methodology can also be applied to all other types of systems, regardless of their structure (e.g., crosslinked polymers: thermosets and elastomers), with proper identification of the components and corresponding parameters.

## 5. Summary and Conclusions

One of the major concerns for using polymers is their sustainability, as general trends lean towards lower material consumption, efficient processing and extreme utilization. Although the sustainability of such polymer-based structures may be evaluated in several ways, simulating their behavior in a virtual world offers several advantages over real-time testing. To predict such behavior, linear and nonlinear viscoelastic laws are considered; however, both approaches are insufficient to provide accurate theoretical or numerical solutions as they are unable to determine residual strains. Regardless of their origin, their accumulation leads to premature failure of the material or structure.

Within the present research, Schapery’s nonlinear viscoelastic model was extended by introducing two components of irrecoverable deformation, displaying material flow (structure related irrecoverable process) and viscoplastic behavior (load related irrecoverable process), accompanied by an analytical and experimental methodology for model evaluation. The investigation was carried out on two versatile and well-established thermoplastic polymers with different morphological structures, i.e., amorphous ABS exhibiting notable flow and semi-crystalline POM, for which flow can be neglected within the considered time–temperature scale. With the use of an advanced modular rheological system, creep and creep recovery tests were conducted in linear and nonlinear domain at temperatures of 70 °C (max. operating temperature) in which materials display both irrecoverable processes. Introducing multi-scale analysis allows parameter identification and evaluation of pure linear and nonlinear viscoelastic, as well as viscoplastic behavior enabling the study of their contribution to overall material response. The evaluation results show extremely accurate predictions as well as exceptional agreement with experimental data displayed with NRMSE<5% for both studied materials, ranging from infinitesimally small to relatively high magnitudes of loading (from 0.1 to 15 MPa of shear stress). Furthermore, the procedure allows the identification of irrecoverable effects even within the linear viscoelastic domain. Hence viscoplastic strains are not associated with yield phenomena (associated with progressive/damaging mechanisms) as often assumed in the literature but rather provide an explanation of the material’s inability to return to its original state.

The proposed experimental and theoretical framework was primarily developed to improve the sustainability and safety of polymers and polymer-based systems under realistic conditions; however, with the appropriate identification of material structures, behavioral components and parameters, it can be a powerful tool to simulate the behavior of other molecular systems, from synthetic to the ones found in nature.

## Figures and Tables

**Table 1:** Summary of processing conditions during three-step manufacturing procedure of amorphous ABS and semi-crystalline POM samples.

Process	Process	ABS	POM
Drying	Drying time	4 h	4 h
	Drying temperature	100 °C	100 °C
Melt mixing	Mixing time	5 min	5 min
	Mixing temperature	240 °C	210 °C
	Screw speed (co-rotating)	50 rpm	50 rpm
Injection molding	Barrel temperature	240 °C	210 °C
	Mold temperature	70 °C	105 °C
	Injection pressure	3 MPa	5 MPa
	Injection time	10 s	10s
	Holding pressure	6 MPa	8 MPa
	Holding time	10 s	10 s

**Table 2:** Nonlinear viscoelastic–viscoplastic model parameters for amorphous ABS and semi-crystalline POM.

Amorphous ABS										
Linear viscoelastic behavior	$J_{0},\;[1/MPa]$		$\phi_{flow},[1/MPa\cdot s]$			$\log\lambda_{i},\;[s]$			$\log J_{i},\;[1/MPa]$	
	$1.28\times 10^{-3}$		$9.37\times 10^{-9}$			−0.5			−7	
						0			−6.04	
						0.5			−4.99	
						1			−4.72	
						1.5			−4.71	
						2			−4.59	
						2.5			−4.46	
						3			−4.33	
						3.5			−4.15	
						4			−4.04	
						4.5			−3.56	
Nonlinear viscoelastic behavior	τ, [MPa]	$g_{0},[/]$			$g_{1},[/]$		$g_{2},[/]$			$a_{\tau },[/]$
	0.1	1			1		1			1
	1	1			1		1			1
	2.5	1			1		1			1
	5	1.04			0.91		1.09			1
	7.5	1.07			0.88		1.14			1
	10	1.14			0.79		1.25			1
	12.5	1.17			0.75		1.33			1
	15	1.20			0.7		1.46			1
Viscoplastic behavior	C,[1/MPa·s]			N,[/]				n,[/]		
	$3.17\times 10^{-7}$			7.26				0.33		
**Semi-crystalline POM**										
Linear viscoelastic behavior	$J_{0},\;[1/MPa]$		$\phi_{flow},[1/MPa\cdot s]$			$\log\lambda_{i},\;[s]$			$\log J_{i},\;[1/MPa]$	
	$1.94\times 10^{-3}$		/			−0.5			−6.99	
						0			−4.09	
						0.5			−3.89	
						1			−3.77	
						1.5			−3.69	
						2			−3.67	
						2.5			−3.70	
						3			−3.79	
						3.5			−3.82	
						4			−3.79	
						4.5			−3.61	
Nonlinear viscoelastic behavior	τ, [MPa]	$g_{0},[/]$			$g_{1},[/]$		$g_{2},[/]$			$a_{\tau },[/]$
	0.1	1			1		1			1
	1	1			1		1			1
	2.5	1			1		1			1
	5	1.05			0.97		1.12			1
	7.5	1.13			0.95		1.18			1
	10	1.25			0.95		1.32			1
	12.5	1.35			0.95		1.39			1
	15	1.40			095		1.48			1
Viscoplastic behavior	C,[1/MPa·s]			N,[/]				n,[/]		
	$2.41\times 10^{-7}$			6.59				0.33		

## Data Availability

The raw data supporting the conclusions of this article will be made available by the authors on request.

## Abbreviations

The following abbreviations are used in this manuscript:

ABS	Acrylonitrile Butadiene Styrene
POM	Polyoxymethylene
TC	Thermal conditioning
TD	Thermo-dynamic state
DSC	Differential dynamic calorimetry
DTMA	Dynamic Thermo-Mechanical Analysis
VE	Viscoelastic
VP	Viscoplastic

## References

[1] European Commission A New Circular Economy Action Plan (2020). EUR-Lex-52020DC0098-EN. https://eur-lex.europa.eu/legal-content/SL/PIN/?uri=CELEX:52020DC0098.

[2] European Commission Ecodesign for Sustainable Product Regulation (2024). Regulation-EU-2024/1781-EN-EUR-Lex. https://eur-lex.europa.eu/eli/reg/2024/1781/oj/eng.

[3] Oseli A., Aulova A., Gergesova M., Emri I. (2020). Effect of temperature on mechanical properties of polymers. Encyclopedia of Continuum Mechanics.

[4] Aulova A., Oseli A., Bek M., Prodan T., Emri I. (2020). Effect of pressure on mechanical properties of polymers. Encyclopedia of Continuum Mechanics.

[5] Emri I., Gergesova M. (2010). Time dependent behavior of solid polymers. Encyclopedia of Life Support Systems.

[6] Brinson H.F., Brinson L.C. (2010). Nonlinear viscoelasticity. Polymer Engineering Science and Viscoelasticity: An Introduction.

[7] Schapery R.A. (1997). Nonlinear viscoelastic and viscoplastic constitutive equations based on thermodynamics. Mech. Time-Depend. Mater..

[8] Schapery R. (1999). Nonlinear viscoelastic and viscoplastic constitutive equations with growing damage. Int. J. Fract..

[9] Tschoegl N.W. (2012). The Phenomenological Theory of Linear Viscoelastic Behavior: An Introduction.

[10] Knauss W.G., Emri I., Lu H. (2008). Mechanics of polymers: Viscoelasticity. Springer Handbook of Experimental Solid Mechanics.

[11] Yannas I.V. (1974). Nonlinear viscoelasticity of solid polymers (in uniaxial tensile loading). J. Polym. Sci. Macromol. Rev..

[12] Yannas I.V., Lunn A.C. (1970). Transition from linear to nonlinear viscoelastic behavior. Part 1 Creep of polycarbonate. J. Macromol. Sci. Part B.

[13] Yannas I.V., Sung N.H., Lunn A.C. (1971). Transition from linear to nonlinear viscoelastic behavior. Part 2. stress relaxation of polycarbonate. J. Macromol. Sci. Part B.

[14] Yannas I.V. (1972). Transition from linear to nonlinear viscoelastic behavior. Part 3 Linearity below and above T_g_. J. Macromol. Sci. Part B.

[15] Starkova O., Aniskevich A. (2007). Limits of linear viscoelastic behavior of polymers. Mech. Time-Depend. Mater..

[16] Jansson J.F. (1973). Studies of relaxation phenomena in polymers. I. The use of periodic square and triangular stress functions. J. Appl. Polym. Sci..

[17] Maxwell B., Guimon C. (1962). Dynamic mechanical spectra and limit of linear viscoelasticity of high polymers. J. Appl. Polym. Sci..

[18] Starkova O., Aniskevich A. (2009). Application of time-temperature superposition to energy limit of linear viscoelastic behavior. J. Appl. Polym. Sci..

[19] Starkova O., Zhang Z., Zhang H., Park H.-W. (2008). Limits of the linear viscoelastic behavior of polyamide 66 filled with TiO_2_ nanoparticles: Effect of strain rate, temperature, and moisture. Mater. Sci. Eng. A.

[20] Knauss W.G., Emri I. (1987). Volume change and the Nonlinearly Thermo-Viscoelastic Constitution of Polymers. Polym. Eng. Sci..

[21] Schapery R.A. (1969). On the characterization of nonlinear viscoelastic materials. Polym. Eng. Sci..

[22] Popelar C.F., Liechti K.M. (2003). A distortion-modified free volume theory for nonlinear viscoelastic behavior. Mech. Time-Depend. Mater..

[23] Chevellard G., Ravi-Chandar K., Liechti K.M. (2012). Modeling the nonlinear viscoelastic behavior of polyurea using a distortion modified free volume approach. Mech. Time-Depend. Mater..

[24] Zink T., Kehrer L., Hirschberg V., Wilhelm M., Böhlke T. (2021). Nonlinear schapery viscoelastic model for thermoplastic polymers. J. Appl. Polym. Sci..

[25] Tong X., Xu J., Doghri I., El Ghezal M.I., Krairi A., Chen X. (2020). A nonlinear viscoelastic constitutive model for cyclically loaded solid composite propellant. Int. J. Solids Struct..

[26] Shim W., Jang J., Choi J.-H., Cho J.-M., Yoon S.-J., Choi C.-H., Yu W.-R. (2020). Simulating rate- and temperature-dependent behaviors of adhesives using a nonlinear viscoelastic model. Mech. Mater..

[27] Nordin L.-O., Varna J. (2006). Methodology for parameter identification in nonlinear viscoelastic material model. Mech. Time-Depend. Mater..

[28] Varna J., Pupure L. (2023). Effect of material state and temperature on nonlinear viscoelastic response: 3D constitutive model and incremental formulation for numerical analysis. Mech. Compos. Mater..

[29] Sun T., Yu C., Yang W., Zhong J., Xu Q. (2020). Experimental and numerical research on the nonlinear creep response of polymeric composites under humid environments. Compos. Struct..

[30] Jafaripour M., Taheri-Behrooz F. (2020). Creep behavior modeling of polymeric composites using Schapery model based on micromechanical approach. Eur. J. Mech./A Solids.

[31] Yang J., Ma X., Wang H., Shang F., Hou D. (2023). Characterization of nonlinear viscoelastic behaviors of CF/EP laminates with consideration to physical aging effect under thermos-mechanical loading. Mech. Mater..

[32] Ihuaenyi R.C., Deng J., Bae C., Xiao X. (2023). An orthotropic nonlinear thermoviscoelastic model for polymeric battery separators. J. Electrochem. Soc..

[33] Oseli A., Mihelčič M., Šobak M., Perše L.S. (2024). Nonlinear time-dependent behavior of rheodictic polymers: A theoretical and experimental investigation. Polym. Test..

[34] Perzyna P. (1971). Themodynamic theory of viscoplasticity. Adv. Appl. Mech..

[35] Takaoka H., Sakaue K. (2020). Evaluation of viscoelastic-viscoplastic characteristics and finite element analyses for thermoplastics. Adv. Compos. Mater..

[36] Chen Y., Smith L.V. (2021). A nonlinear viscoelastic–viscoplastic model for adhesives. Mech. Time-Depend. Mater..

[37] Haddad M., Doghri I., Pierard O. (2022). Viscoelastic-viscoplastic polymer composites: Development and evaluation of two very dissimilar mean-field homogenization models. Int. J. Solids Struct..

[38] Zapas L., Crissman J. (1984). Creep and recovery behaviour of ultra-high molecular weight polyethylene in the region of small uniaxial deformations. Polymer.

[39] Nordin L.-O., Varna J. (2006). Nonlinear viscoplastic and nonlinear viscoelastic material model for paper fiber composites in compression. Compos. Part A Appl. Sci. Manuf..

[40] Ferry J.D. (1980). Viscoelastic Properties of Polymers.

[41] Zupančič B., Emri I. (2009). Time-dependent constitutive modeling of drive belts—II. The effect of the shape of material retardation spectrum on the strain accumulation process. Mech. Time-Depend. Mater..

[42] Park S., Schapery R. (1999). Methods of interconversion between linear viscoelastic material functions. Part I-a numerical method based on Prony series. Int. J. Solids Struct..

[43] Emri I., Tschoegl N.W. (1995). Determination of mechanical spectra from experimental responses. Int. J. Solids Struct..

[44] Roths T., Maier D., Friedrich C., Marth M., Honerkamp J. (2000). Determination of the relaxation time spectrum from dynamic moduli using an edge preserving regularization method. Rheol. Acta.

[45] Struik L.C.E. (1977). Physical aging in plastics and other glassy materials. Polym. Eng. Sci..

[46] Oseli A., Prodan T., Susič E., Perše L.S. (2020). The effect of short fiber orientation on long term shear behavior of 40% glass fiber reinforced polyphenylene sulfide. Polym. Test..

[47] (2023). Plastics—Differential Scanning Calorimetry.

[48] (2019). Plastics—Determination of Dynamic Mechanical Properties.

[49] Mark J.E. (2007). Physical Properties of Polymers Handbook.

[50] Menard K.P., Menard N.R. (2008). Dynamic Mechanical Analisys: A Practical Introduction.

[51] Wang J., Xu Y., Zhang W., Moumni Z. (2016). A damage-based elastic-viscoplastic constitutive model for amorphous glassy polycarbonate polymers. Mater. Des..

[52] Aoyagi Y., Camboulives L.N. (2025). Modeling of nonlinear viscoelastic-viscoplastic behavior of glassy polymers based on intramolecular rotation of molecular chains. Int. J. Plast..

[53] Huber-Carol C., Balakrishnan N., Nikulin M.S., Mesbah M. (2002). Goodness-of-Fit Tests and Model Validity.

